# Pancreatic fistula after pancreatoduodenectomy due to compression of the superior mesenteric vessels: a case report

**DOI:** 10.1186/s12893-020-00828-2

**Published:** 2020-07-28

**Authors:** Hanteng Yang, Yanxian Ren, Zeyuan Yu, Huinian Zhou, Shuze Zhang, Changjiang Luo, Zuoyi Jiao

**Affiliations:** 1 Department of General Surgery, Lanzhou University Second Hospital, No.82 Cuiyingmen, Lanzhou, 730030 Gansu China; 2 Cuiying Biomedical Research Center, Lanzhou University Second Hospital, No.82 Cuiyingmen, Lanzhou, 730030 Gansu China

**Keywords:** Pancreatic fistula, Pancreaticoduodenectomy, Pancreaticojejunostomy, Superior mesenteric vessel

## Abstract

**Background:**

Pancreatic fistula is a common complication after pancreaticoduodenectomy, which could be caused by: soft pancreatic tissue, pancreatic duct diameter < 3 mm and body mass index ≥25 kg/m^2^. Here we report a case of pancreatic fistula due to obstruction of the jejunal loop due to compression of the jejunal loop by the superior mesenteric vessels.

**Case presentation:**

A 68-year-old man was admitted to our ward due to intermittent epigastric distension and pain. After various examinations and treatments, he was diagnosed with middle bile duct cancer. Pancreaticoduodenectomy was performed, and pancreaticojejunostomy and hepaticojejunostomy were completed by lifting the jejunal loop from behind the superior mesenteric vessels to the upper region of the colon. On postoperative day 9, the patient developed acute diffuse peritonitis, and on postoperative day 10, the patient underwent a second exploratory laparotomy, during which it was confirmed that the pancreatic fistula was caused by obstruction of the jejunal loop due to compression of the jejunal loop by the superior mesenteric vessels, then the patient recovered and was discharged alive after retrograde drainage in the jejunum.

**Conclusions:**

The superior mesenteric vessels after pancreaticoduodenal surgery can compress the jejunal loop and cause obstruction leading to serious complications, and it is recommended that general surgeons should avoid lifting the jejunal loop from the posterior aspect of the superior mesenteric vessels to complete the anastomosis.

## Background

Pancreatoduodenectomy (PD) is advocated for treating most malignant and benign neoplasms of the pancreatic head and periampullary region [1]. PD procedures can be performed in high-volume medical centers with a mortality rate of < 5% and an up to 50% risk of perioperative complications [2, 3]. Postoperative pancreatic fistula (POPF) is a common complication of pancreaticojejunostomy and its occurrence remains considerable, ranging from 13 to 41% [4, 5]. The occurrence of a clinically relevant (CR)-POPF has been advocated as a trigger factor for developing secondary complications, such as post-pancreatectomy hemorrhage, infections, and postoperative failure to thrive [6].

Several factors contribute to the development of a POPF, and every pancreatic surgeon must be aware of these factors. Patient-related factors can influence POPF development, including soft pancreatic tissue, a small pancreatic duct diameter of < 3 mm, and a body mass index ≥25 kg/m^2^, and have been proven to be independent risk factors for POPF [7–10]. Moreover, technical and perioperative factors also contribute to the development of POPF.

In this report, we present one case of Grade-C POPF after pancreaticojejunostomy due to the completion of anastomosis in the upper mesenteric vessels behind the jejunum loop obstruction extraction, along with a review of the literature.

## Case presentation

**Fig. 1:**
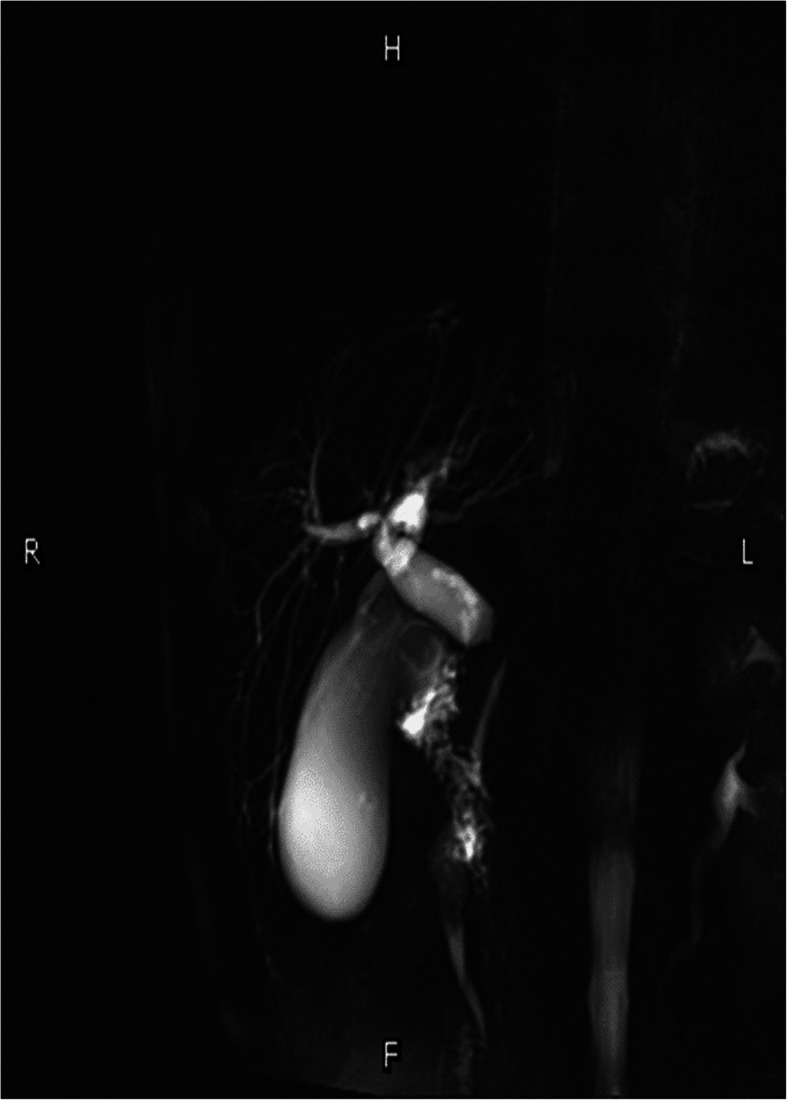
MRI showed local wall thickens and enhanced in the supper pancreatic segment of the common bile duct, and lumen is stenosis, mostly likely choledochal carcinoma, with upper bile duct dilatation

**Fig. 2:**
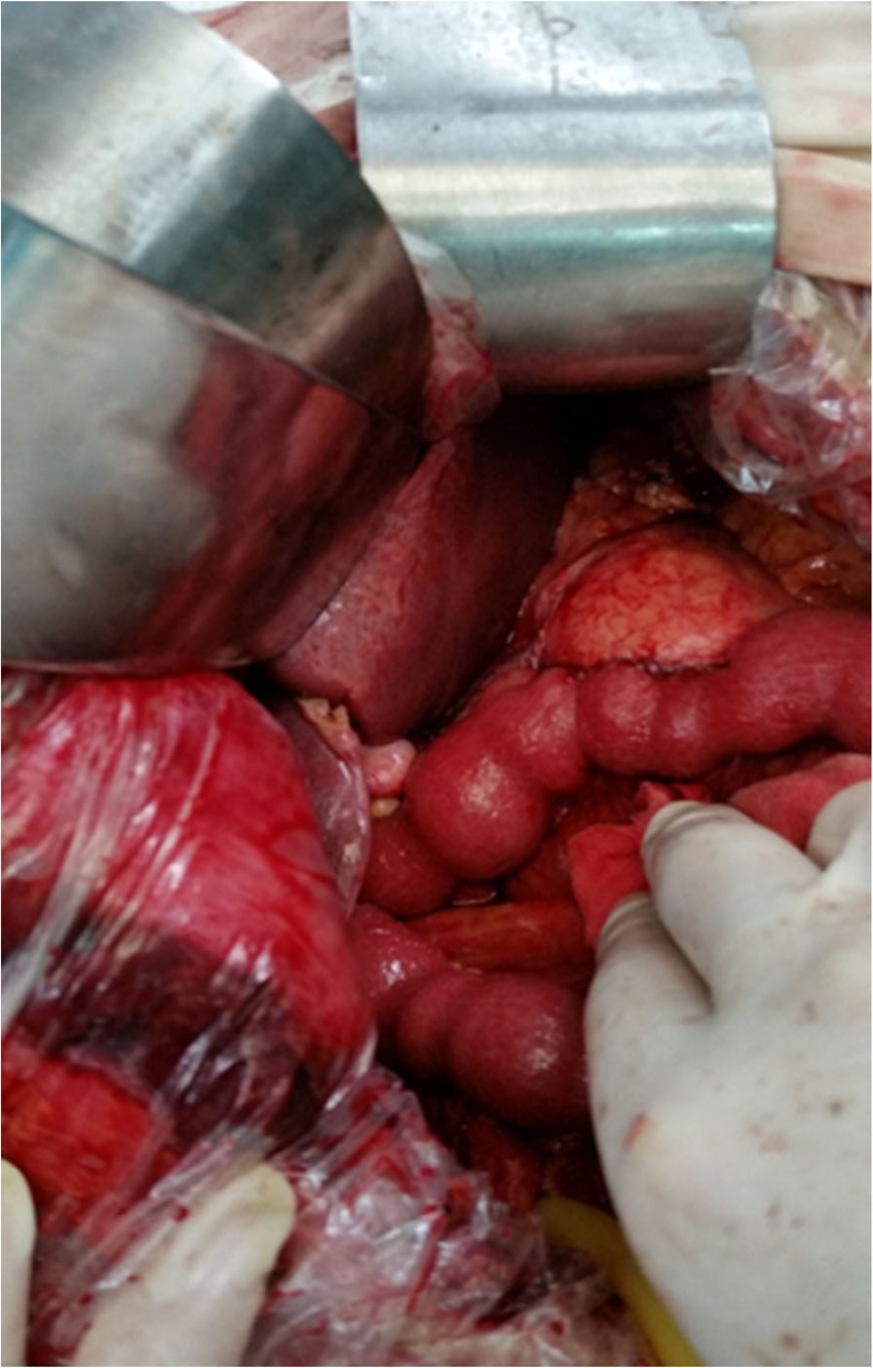
Pancreaticojejunostomy at completion of surgery

A 68-year-old man was admitted to our ward with intermittent epigastric distention for more than 1 month. The patient underwent surgery for penile cancer 21 years ago. The physical examination was unremarkable, with the exception of slight tenderness in the right upper abdomen. The patient’s height was 175 cm, weight was 70 kg, and body mass index (BMI) was 22.86. The initial laboratory results showed: total bilirubin (TBIL) 17.9 umol/L, direct bilirubin (DBIL) 9.3 umol/L, alanine aminotransferase (ALT) 526.5 U/L, aspartic aminotransferase (AST) 302.1 U/L, alkaline phosphatase 1756.6 U/L, glutamyl transpeptidase (GGT) 2240.6 U/L, and carbohydrate 19–9 (CA19–9) 26.11 u/ml. The initial leucocyte count, platelet count, renal function, coagulation profile, and other electrolytes were normal. Upper abdominal magnetic resonance imaging (MRI) showed local wall thickening enhanced in the upper pancreatic segment of the common bile duct and lumen stenosis, mostly considering choledochal carcinoma, with upper bile duct dilation and without evidence of invasion of the superior mesenteric vessels and metastasis (Fig. 1). A malignant tumor of the common bile duct was diagnosed preoperatively. Due to the elevated level of ALT in the patient, reduced glutathione was administered to protect the liver function. However, a week later, the ALT level was 840.6 U/L; thus, endoscopic retrograde cholangiopancreatography (ERCP) and nasal bile duct drainage were performed. Four days after ERCP, the ALT and AST levels decreased to 840.6 U/L and 38.4 U/L, respectively, and PD was performed on the fifth day after ERCP. The distal stomach, gallbladder, common bile duct, pancreatic head, and a small part of the jejunum were removed. The distal jejunum was lifted up behind the superior mesenteric vessels to the superior colonic region, then pancreaticojejunostomy, chojejunostomy, gastrojejunostomy, and Braun jejunostomy were completed successively. The pancreaticojejunostomy was conducted using the duct-to-mucosa method (Fig. 2), and the chojejunostomy, gastrojejunostomy, and Braun jejunostomy were performed using the running suturing technique. Intraoperative findings revealed soft pancreatic texture and pancreatic duct was 3 mm.

**Fig. 3:**
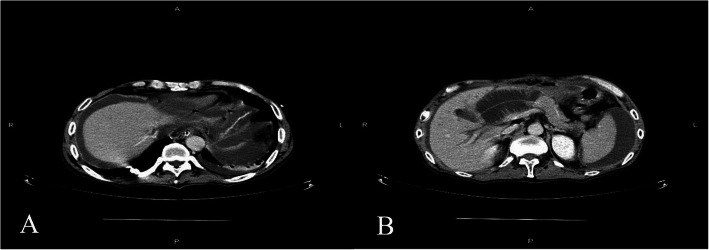
On POD 10, CT showed (**a**) bilateral subphrenic and intraperitoneal free gas and abdominal pelvic effusion; (**b**) dilatation and effusion of upper abdominal intestine

On postoperative day (POD) 1, the endotracheal intubation was removed and gastrointestinal function was restored. A liquid diet was then given on POD 5 and a semi-liquid diet was given on POD 7. During this process, the vital signs and drainage amylase test results of the patient were normal. On POD 9, the patient suddenly experienced severe epigastric pain accompanied by nausea and vomiting. Physical examination revealed tension in the abdominal muscles, tenderness throughout the abdomen, and rebound pain. The patient’s heart rate was 130 beats/minute, blood pressure (BP) was 106/67 mmHg, leucocyte count was 8.25 × 10^9^ cells/L, and drainage amylase level was 21.8 U/L. Emergency total abdominal computed tomography (CT) showed a small amount of fluid in the abdominal and pelvic cavities, a dilated bowel, and effusion in the upper abdominal bowel cavity, which was considered to be an obstruction. The patient was admitted to the intensive care unit and received conservative treatment, including fasting, gastrointestinal decompression, anti-infective treatment, proton pump inhibitors, somatostatin, and analgesia. On POD 10, the heart rate was 170 beats/minute, BP was 108/59 mmHg, leucocyte count was 13.15 × 10^9^ cells/L, and drainage amylase level was 145.5 U/L. Reexamination using total abdominal CT suggested bilateral subdiaphragmatic free gas, abdominal and pelvic effusion, anastomotic leakage (Fig. 3a), expansion of the upper abdominal bowel, effusion, and obstruction (Fig. 3b). Subsequently, the patient appeared to be unconscious, and he was given assisted breathing by endotracheal intubation ventilation, continuous renal replacement therapy (CRRT), and peritoneal puncture and drainage under ultrasound guidance. About 1000 ml of yellow-green fluid was extracted, and the level of amylase in the drainage fluid was 896.3 U/L.

**Fig. 4:**
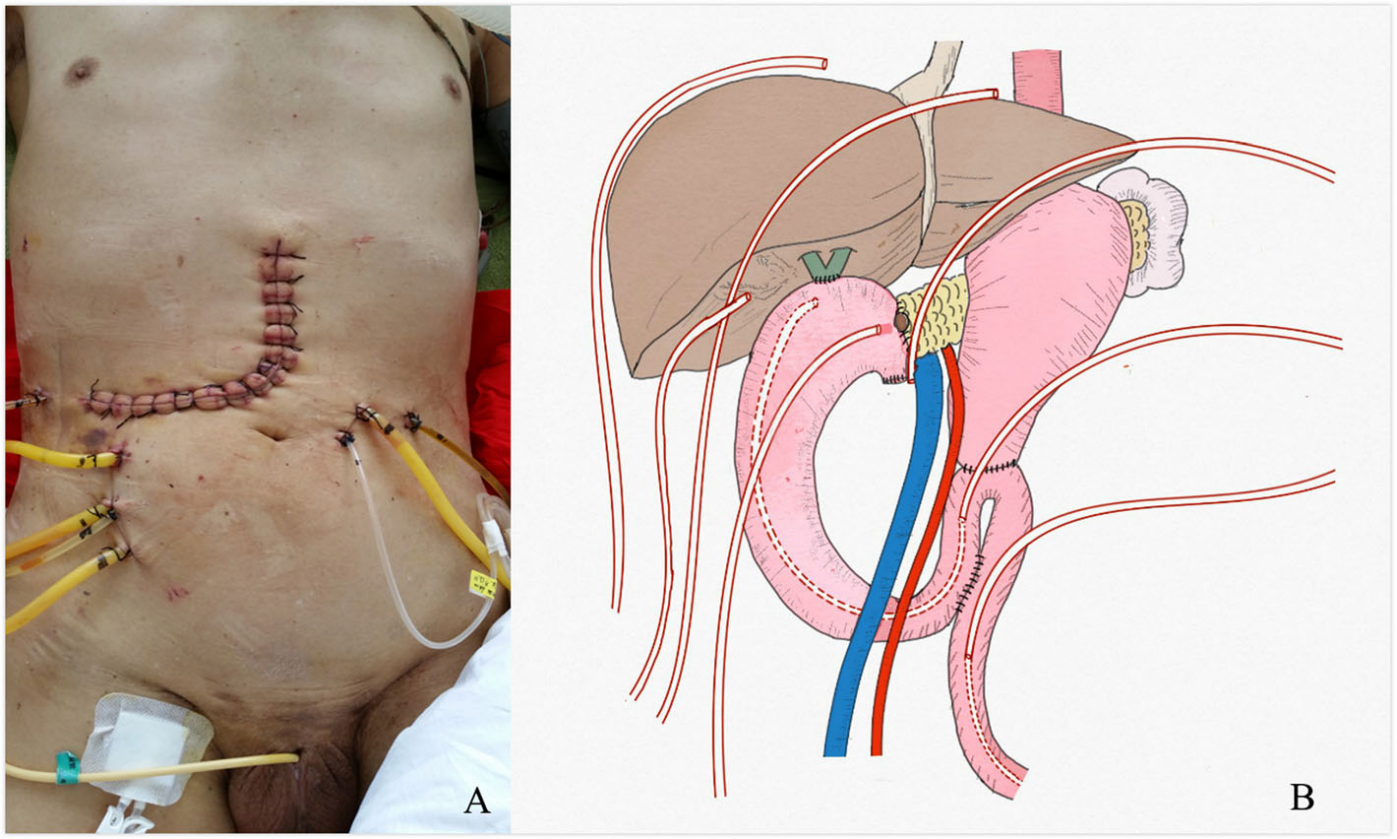
(**a**) Abdominal incision and drainage tube after secondary operation;(**b**) Schematic diagram of drainage tube in secondary operation

Considering anastomotic leakage, acute diffuse peritonitis, and septic shock, the patient underwent a second exploratory laparotomy. During the operation, the superior mesenteric vessels were found to be compressing the jejunum, resulting in obvious dilation of the jejunum loop in the upper colon region, and the jejunum in the lower colon region was empty. In front of the pancreatojejunostomy location, there was a leak with a diameter of about 5 mm, and a large amount of fluid surrounded the liver and spleen. A retrograde gastric tube was implanted in the jejunum from the proximal end of the Braun anastomosis to near the chojejunostomy location, a nutrition tube was implanted in the distal jejunum under the Braun anastomosis, several silicone drainage tubes were placed around the liver, spleen, and pelvis, and the operation was completed (Fig. 4a and Fig. 4b).

On the post-second operative day (PSOD) 1, the patient’s temperature was 37.9 °C, pulse rate was 90 times/minute, BP was 120/70 mmHg, and the endotracheal tube was removed after the patient awoke. Following that, enteral nutrition was performed, peritoneal lavage and drainage were continued, and anti-infective agents, proton pump inhibitors, somatostatin, and other treatments were continued. On PSOD 32, the patient’s pancreatic fistula was healed and the abdominal drainage tube was removed. The patient was discharged on PSOD 35. On PSOD 45, the nutrition tube was removed and the gastric tube in the jejunum loop was removed, indicating full recovery of the patient.

## Discussion and conclusion

Thanks to advances in surgical techniques and perioperative management, the current mortality rate after PD procedures has decreased to less than 5% in high-volume centers [11, 12], but the postoperative morbidity remains high with a complication rate of at least 45% [13, 14]. Pancreatic anastomosis is the “Achilles heel” of pancreatic resection and POPF, and is a common complication following PD procedures [15]. The spectrum of POPF cases can be mild to severe. The International Study Group of Pancreatic Surgery (ISGPS) stratifies POPFs into three risk grades [16]. Grade A POPF is mild and of no clinical importance, while Grade B POPF mandates a change in postoperative management or requires prolonged drainage for more than 3 weeks post-surgery, potentially increasing the incidence of infection. Grade C POPF can lead to acute hemorrhage and abdominal sepsis, increasing both morbidity and mortality.

It is known that several factors contribute to the development of a POPF. There are technical and perioperative factors, on the one hand, and patient-related factors, on the other hand [17]. In 2013, Callery et al. identified several perioperative factors related to the occurrence of POPF, such as soft pancreatic tissue, a small pancreatic duct diameter of < 3 mm, and a body mass index ≥25 kg/m^2^ [6]. Other less investigated patient factors, such as body composition parameters (i.e. sarcopenia, obesity, and the combination of these two parameters) [18, 19], and perioperative hyperhydration have been associated with a higher risk of CR-POPF [20, 21].

A recent meta-analysis [22] comparing duct-to-mucosa anastomosis and invagination pancreatojejunostomies showed that the rate of POPF was approximately 20% without significant differences between the two reconstruction methods. However, some non-randomized, retrospective studies have recently reported the safety and feasibility of Blumgart anastomosis, a pancreaticojejunostomy method, showing its low postoperative mortality rate (1–3%), reoperation rate (5–7%), and acceptable POPF rate (15–20%). Furthermore, these studies have shown that Blumgart anastomosis is more effective with respect to other pancreatic anastomoses, minimizing severe complications [23–26]. However, all these studies were retrospective, their impact on the clinical evidence was poor, and they require confirmation by a randomized controlled trial.

In this case, we retrospectively found that the occurrence of pancreatic leakage was due to technical defects. The pancreaticojejunostomy was completed following the lifting of the jejunum loop behind the superior mesenteric vessels, the superior mesenteric vessels compressed the loop, causing obstruction of the loop, and the pressure in the intestinal cavity of the loop was increased due to the accumulation of digestive fluid, eventually leading to rupture of the pancreaticojejunostomy. Fortunately, laparotomy was performed actively and a retrograde gastric tube was implanted in the jejunum from the proximal end of the Braun anastomosis to near the chojejunostomy location, playing a positive role in the decompression of the jejunal loop. A nutritional tube was implanted into the distal jejunum to address the patient’s enteral nutrition problems. Eventually, the patient recovered and has survived to this day.

In pancreaticoduodenectomy, lifting the distal jejunum behind the superior mesenteric vessels to complete the pancreaticojejunostomy and choledochojejunostomy could lead to possible risk of jejunal loop obstruction due to compression of the superior mesenteric vessels and may also cause serious consequences such as pancreaticojejunostomy rupture or choledochojejunostomy rupture, therefore, surgeons should avoid lifting the jejunal loop behind the superior mesenteric vessels. Grade C POPF after pancreaticojejunostomy is a serious challenge for pancreatic surgeons. Various factors that contribute to POPF development have been reported in the previous literature, but there is no report that compression of the superior mesenteric vessels on the loop of the jejunum leads to POPF. This case may serve as a warning to pancreatic surgeons.

## Data Availability

The data supporting the findings of this study are available within the article.

## Abbreviations

PD: Pancreatoduodenectomy
CR-POPF: Clinically relevant postoperative pancreatic fistula
TBIL: Total bilirubin
DBIL: Direct bilirubin
ALT: Alanine aminotransferase
AST: Aspartic aminotransferase
GGT: Glutamyl transpeptidase
MRI: Magnetic resonance imaging
ERCP: Endoscopic retrograde cholangiopancreatography
POD: Postoperative day
PSOD: Post-second operative day
CT: Computed tomography

## References

[1] Xingjun G, Feng Z, Meiwen Y, Jianxin J, Zheng H, Jun G, Tao H, Rui Z, Leida Z, Min W (2019). A score model based on pancreatic steatosis and fibrosis and pancreatic duct diameter to predict postoperative pancreatic fistula after Pancreatoduodenectomy. BMC Surg.

[2] Kitahata Y, Hirono S, Kawai M, Okada KI, Miyazawa M, Shimizu A, Kobayashi R, Ueno M, Hayami S, Shimokawa T (2018). Intensive perioperative rehabilitation improves surgical outcomes after pancreaticoduodenectomy. Langenbeck's Arch Surg.

[3] Narayanan S, Martin AN, Turrentine FE, Bauer TW, Adams RB, Zaydfudim VM (2018). Mortality after pancreaticoduodenectomy: assessing early and late causes of patient death. J Surg Res.

[4] Winter JM, Cameron JL, Campbell KA, Arnold MA, Chang DC, Coleman J, Hodgin MB, Sauter PK, Hruban RH, Riall TS (2006). 1423 pancreaticoduodenectomies for pancreatic cancer: a single-institution experience. J Gastrointest Surg.

[5] Nahm CB, Connor SJ, Samra JS, Mittal A (2018). Postoperative pancreatic fistula: a review of traditional and emerging concepts. Clin Exp Gastroenterol.

[6] Angrisani M, Sandini M, Cereda M, Paiella S, Capretti G, Nappo G, Roccamatisi L, Casciani F, Caccialanza R, Bassi C, et al. Preoperative adiposity at bioimpedance vector analysis improves the ability of fistula risk score (FRS) in predicting pancreatic fistula after pancreatoduodenectomy. Pancreatology. 2020;20(3):545–50. 10.1016/j.pan.2020.01.008 31980350

[7] Belyaev O, Munding J, Herzog T, Suelberg D, Tannapfel A, Schmidt WE, Mueller CA, Uhl W (2011). Histomorphological features of the pancreatic remnant as independent risk factors for postoperative pancreatic fistula: a matched-pairs analysis. Pancreatology.

[8] Eshmuminov D, Schneider MA, Tschuor C, Raptis DA, Kambakamba P, Muller X, Lesurtel M, Clavien PA (2018). Systematic review and meta-analysis of postoperative pancreatic fistula rates using the updated 2016 international study group pancreatic fistula definition in patients undergoing pancreatic resection with soft and hard pancreatic texture. HPB (Oxford).

[9] Hackert T, Buchler MW (2015). Management of postoperative pancreatic fistula. Chirurg.

[10] Seeliger H, Christians S, Angele MK, Kleespies A, Eichhorn ME, Ischenko I, Boeck S, Heinemann V, Jauch KW, Bruns CJ (2010). Risk factors for surgical complications in distal pancreatectomy. Am J Surg.

[11] Ghaferi AA, Birkmeyer JD, Dimick JB (2009). Variation in hospital mortality associated with inpatient surgery. N Engl J Med.

[12] Kimura W, Miyata H, Gotoh M, Hirai I, Kenjo A, Kitagawa Y, Shimada M, Baba H, Tomita N, Nakagoe T (2014). A pancreaticoduodenectomy risk model derived from 8575 cases from a national single-race population (Japanese) using a web-based data entry system: the 30-day and in-hospital mortality rates for pancreaticoduodenectomy. Ann Surg.

[13] Lubrano J, Bachelier P, Paye F, Le Treut YP, Chiche L, Sa-Cunha A, Turrini O, Menahem B, Launoy G, Delpero JR (2018). Severe postoperative complications decrease overall and disease free survival in pancreatic ductal adenocarcinoma after pancreaticoduodenectomy. Eur J Surg Oncol.

[14] Shrikhande SV, Sivasanker M, Vollmer CM, Friess H, Besselink MG, Fingerhut A, Yeo CJ, Fernandez-delCastillo C, Dervenis C, Halloran C (2017). Pancreatic anastomosis after pancreatoduodenectomy: a position statement by the International Study Group of Pancreatic Surgery (ISGPS). Surgery.

[15] Li T, D’Cruz RT, Lim SY, Shelat VG. Somatostatin analogues and the risk of post-operative pancreatic fistulas after pancreatic resection - A systematic review & meta-analysis. Pancreatology. 2020;20(3):545–50. 10.1016/j.pan.2019.12.015 31980352

[16] Bassi C, Marchegiani G, Dervenis C, Sarr M, Abu Hilal M, Adham M, Allen P, Andersson R, Asbun HJ, Besselink MG (2017). The 2016 update of the International Study Group (ISGPS) definition and grading of postoperative pancreatic fistula: 11 years after. Surgery.

[17] Luu AM, Krasemann L, Fahlbusch T, Belyaev O, Janot-Matuschek M, Uhl W, Braumann C. Facing the surgeon's nightmare: incidence and management of postoperative pancreatic fistulas grade C after pancreaticoduodenectomy based on the updated definition of the international study Group of Pancreatic Surgery (ISGPS). J Hepatobiliary Pancreat Sci. 2020;27(4):171–81. 10.1002/jhbp.713 31951086

[18] Amini N, Spolverato G, Gupta R, Margonis GA, Kim Y, Wagner D, Rezaee N, Weiss MJ, Wolfgang CL, Makary MM (2015). Impact total psoas volume on short- and long-term outcomes in patients undergoing curative resection for pancreatic adenocarcinoma: a new tool to assess sarcopenia. J Gastrointest Surg.

[19] Sandini M, Bernasconi DP, Ippolito D, Nespoli L, Baini M, Barbaro S, Fior D, Gianotti L (2015). Preoperative computed tomography to predict and stratify the risk of severe pancreatic fistula after pancreatoduodenectomy. Medicine (Baltimore).

[20] Sandini M, Fernandez-Del Castillo C, Ferrone CR, Ruscic KJ, Eikermann M, Warshaw AL, Lillemoe KD, Qadan M (2019). Intraoperative fluid administration and surgical outcomes following pancreaticoduodenectomy: external validation at a tertiary referral center. World J Surg.

[21] Wang S, Wang X, Dai H, Han J, Li N, Li J (2014). The effect of intraoperative fluid volume administration on pancreatic fistulas after pancreaticoduodenectomy. J Investig Surg.

[22] Lyu Y, Li T, Wang B, Cheng Y, Zhao S (2018). Selection of pancreaticojejunostomy technique after pancreaticoduodenectomy: duct-to-mucosa anastomosis is not better than invagination anastomosis: a meta-analysis. Medicine (Baltimore).

[23] Hirono S, Kawai M, Okada KI, Miyazawa M, Kitahata Y, Hayami S, Ueno M, Yamaue H (2019). Modified Blumgart mattress suture versus conventional interrupted suture in pancreaticojejunostomy during pancreaticoduodenectomy: randomized controlled trial. Ann Surg.

[24] Kawakatsu S, Inoue Y, Mise Y, Ishizawa T, Ito H, Takahashi Y, Saiura A (2018). Comparison of pancreatojejunostomy techniques in patients with a soft pancreas: Kakita anastomosis and Blumgart anastomosis. BMC Surg.

[25] Li YT, Zhang HY, Xing C, Ding C, Wu WM, Liao Q, Zhang TP, Zhao YP, Dai MH (2019). Effect of Blumgart anastomosis in reducing the incidence rate of pancreatic fistula after pancreatoduodenectomy. World J Gastroenterol.

[26] Casadei R, Ricci C, Ingaldi C, Alberici L, De Raffele E, Minni F. Comparison of Blumgart anastomosis with duct-to-mucosa anastomosis and invagination pancreaticojejunostomy after Pancreaticoduodenectomy: a single-center propensity score matching analysis. J Gastrointest Surg. 2020. 10.1007/s11605-020-04528-3 31997074

